# Anti-nociceptive properties in rodents and the possibility of using polyphenol-rich fractions from *sida urens* L. (Malvaceae) against of dental caries bacteria

**DOI:** 10.1186/1476-0711-12-14

**Published:** 2013-06-21

**Authors:** Kiessoun Konaté, Patrice Zerbo, Maurice Ouédraogo, Crépin I Dibala, Hilou Adama, Oksana Sytar, Marian Brestic, Nicolas Barro

**Affiliations:** 1 Unit of Formation in Sciences Applied and Technological (UFR/SAT) and Institute of Sciences of the Environment and the Rural Development (ISEDR), Polytechnic University of Dédougou, Dédougou, Burkina Faso; 2 Laboratory of Plant Ecology and Biology, University of Ouagadougou, 09 PO Box: 848, Ouagadougou 09, Burkina Faso; 3 Laboratory of Animal Physiology, University of Ouagadougou, 09 PO Box: 848, Ouagadougou 09, Burkina Faso; 4 Laboratory of Food Biochemistry, Enzymology, Biotechnology and Bioinformatic, University of Ouagadougou, Ouagadougou, Burkina Faso; 5 Laboratory of Biochemistry and Applied Chemistry, University of Ouagadougou, 09 PO Box: 848, Ouagadougou 09, Burkina Faso; 6 Department of Plant Physiolgy and Ecology, Taras Shevchenko National University of Kyiv, Volodymyrs’ka St. 64, 01601, Kyiv, Ukraine; 7 Department of Plant Physiology, Slovak University of Agriculture, Nitra, A. Hlinku 2, 94976, Nitra, Slovak Republic; 8 Laboratory of Biochemistry and Molecular Genetics Microbial, University of Ouagadougou, 03 P.O. Box: 7131, Ouagadougou 03, Burkina Faso

## Abstract

**Background:**

*Sida urens* L. (Malvaceae) is in flora of Asian medicinal herbs and used traditionally in West of Burkina Faso for the treatment of infectious diseases and particularly used against, dental caries bacteria, fever, pain and possesses analgesic properties. This study was conducted to reveal the antibacterial effect against dental caries bacteria on the one hand, and evaluate their analgesic capacity in experimental model with Swiss mice and on the other hand, with an aim to provide a scientific basis for the traditional use of this plant for the management of dental caries bacteria.

**Method:**

The antibacterial assays in this study were performed by using inhibition zone diameters, MIC (Minimum inhibitory concentration) and MBC (Minimal bactericidal concentration) methods. On the whole the dental caries bacteria (Gram-positive and Gram-negative bacterial strains) were used. Negative control was prepared using discs impregnated with 10% DMSO in water and commercially available Gentamicin from Alkom Laboratories LTD was used as positive reference standards for all bacterial strains. In acute toxicity test, mice received doses of extract (acetone/water extract) from *Sida urens* L. by intraperitoneal route and LD_50_ was determined in Swiss mice. As for analgesic effects, acetic acid writhing method was used in mice. The acetic acid-induced writhing method was used in mice with aim to study analgesic effects.

**Results:**

The results showed that the highest antibacterial activities were founded with the polyphenol-rich fractions against all bacterial strains compared to the standard antibiotic. About preliminary study in acute toxicity test, LD_50_ value obtained was more than 5000 mg/kg b.w. Polyphenol-rich fractions produced significant analgesic effects in acetic acid-induced writhing method and in a dose-dependent inhibition was observed.

**Conclusion:**

These results validate the ethno-botanical use of *Sida urens* L. (Malvaceae) and demonstrate the potential of this herbaceous as a potential antibacterial agent of dental caries that could be effectively used for future health care purposes.

## Background

Plants have been one of the important sources of medicines since the beginning of human civilization [1]. Today, nearly 88% of the global population turns to plant derived medicines as they are first line of defence for maintaining health and combating diseases [2].

In effect, dental decay is the most prevalent disease affecting humanity. Teeth get decayed due to a combination of causes that include bad oral hygiene, stagnation of food on or around the teeth, presence of plaque on the tooth structure and the presence of caries causing microorganisms [3]. The presence of a certain types of microorganism was discovered during the last decade in dental plaques. The nucleating role of the microorganisms in the formation of dental calculus shows similarities to that of nanobacteria in calcification [4]. Periodontal disease has long been recognized as a chronic disease, but literature describes it as a disease derived entirely from the effects of a microbial colonization of the gingival crevice. If this were so, it would mean that periodontal disease is unique among chronic diseases, all of which represent the long-term cumulative effects of interaction between a host biologic system and the surrounding environment [5]. Antibiotic resistance is the ability of a microorganism to withstand the effects of an antibiotic. It is a specific type of drug resistance. Antibiotic resistance evolves naturally via natural selection through random mutation, but it could also be engineered by applying an evolutionary stress on a population. Once such a gene is generated, bacteria can then transfer the genetic information in a horizontal fashion (between individuals) by plasmid exchange. The patterns of antibiotic usage greatly affect the number of resistant organisms which develop. Overuse of broad spectrum antibiotics, such as second and third generation greatly hastens the development of resistance. Other factors contributing towards resistance include incorrect diagnosis, unnecessary prescriptions and improper use of antibiotics by patients [6]. Antibiotic resistance in microorganisms recovered from the acute dental abscess has been reported to be increasing in some populations studied over the last few decades. The resistance problem demands that a renewed effort be made to seek antibacterial agents effective against pathogenic bacteria resistant to current antibiotics [5]. One of the possible strategies towards this objective is the rational localization of bioactive phytochemicals [5]. Plants have a limitless ability to synthesize aromatic substances, most of which are phenols or their oxygen substituted derivatives such as tannins. Many of the herbs and spices used by humans to season food yield have useful medicinal compounds including those having antibacterial activity [7]. Plant derived drugs remain an important resource especially in developing countries to combat serious diseases.

Considering the important applications of some plant species belonging to this genus in folk and traditional medicine systems, the present study was designed to investigate the analgesic effect and antibacterial properties of polyphenol-rich fractions from *Sida urens* L. (Malvaceae) against dental caries and their analgesic effect in experimental model.

## Materials

### Identification of plants material

*Sida urens* L. was collected fresh in August 2008 in Gampela, 25 Km east of Ouagadougou, capital of Burkina Faso. The plant was botanically identified by Prof. Millogo-Rasolodimby from the plants Biology Department of the University of Ouagadougou. Voucher specimen was deposited in the Herbarium of the La.B.E.V. (Laboratory of Plant Ecology and Biology, UFR/SVT of University of Ouagadougou) from the University of Ouagadougou.

### Bacterial strains and antibiotics

Microorganisms used in this study were isolated from clinical samples at Laboratory of the General Hospital of Ouagadougou in Burkina Faso. Commercially available antibiotic diffusion discs (10 μg/disc) and Gentamicin were purchased from Alkom Laboratories LTD. Clinical isolates were: *Pseudomonas aeruginosa*, *Streptococcus salivarius*, *Streptococcus viridans*, *Streptococcus mutans*, *Bacillus megaterium*, and *Neisseria catarrhalis*. The following microorganisms were all identified by the use of their biochemical profiles as recommended by [8].

### Chemicals

All reagents were of analytical grade. Acetone was supplied by Fluka chemie (Buchs, Switzerland). INT (p-iodonitrotetrazolium chloride) was purchased from sigma-Aldrich chemie (Steinheim, Germany).

### Animals handling for toxicity test

Swiss NMRI mice (20-30 g) of both sexes were used for this study. All animals were housed in cages under controlled conditions of 12-h light/12-h dark cycle and 25°C. They received pellets food enriched with protein 20% and water *ad libitum*. They were deprived of food for 15h (but with access to drinking water) and weighed before the experiments. Experiments on animals were performed in accordance with the ethical guidelines of the Ethical Committee of laboratory animals for biomedical research approved by the Medical Ethical Committee of “Université de Ouagadougou, Burkina Faso” [9].

## Methods

### Preparation of extracts for acute toxicity study

Fifty grams of powdered plant materials (dried in laboratory condition) was extracted with 500 ml of acetone 80% (400 ml acetone + 100 ml water) for 24 h under mechanic agitation (SM 25 shaker, Edmund BÜHLER, Germany) at room temperature. After filtration, acetone was removed under reduced pressure in a rotary evaporator (BÜCHI, Rotavopor R-200, Switzeland) at approximately 40°C and freeze-dried (Telstar Cryodos 50 freeze-dryer). The extract was weighed before packing in waterproof plastic flasks and stored at 4°C until use.

### Polyphenols extraction

The harvested plant materials fresh (broken into leaf stems) were dried in the laboratory at room temperature (20-25°C), afterwards samples were ground to pass a sieve of 0.3 mm. Polyphenols were extracted with aqueous acetone (80%, v/v). The extract was then washed with hexane to remove chlorophyll and other low molecular weight compounds. Acetone was evaporated and the extract was lyophilized and stored at 22°C prior to biological tests. For the tests, lyophilized sample was dissolved with 10% DMSO in water at the desired concentration [2].

### In vitro antibacterial activity

#### Preparation of inocula

The susceptibility tests were performed by Mueller Hinton agar-well diffusion method [10]. The bacterial strains grown on nutrient agar at 37°C for 18 h were suspended in a saline solution (0.9%, w/v) NaCl and adjusted to a turbidity of 0.5 Mac Farland standard (10^8^ CFU/ml). To obtain the inocula, these suspensions were diluted 100 times in Muller Hinton broth to give 10^6^ colony forming units (CFU)/ml [11].

#### Preparation of discs

The stock solutions of polyphenol-rich fractions from *Sida urens* L., was dissolved in 10% dimethylsulfoxide (DMSO) in water [12] at a final concentration of 4 mg/ml after a serial two-fold dilution. Each stock solution of polyphenol-rich fractions from *Sida urens* L., was sterilized by filtration through 0.22 μm sterilizing Millipore express filter. The sterile discs (6 mm) were impregnated with 10 μL of the sterile solution of polyphenol-rich fractions. Negative controls were prepared using discs impregnated with 10% DMSO in water and commercially available antibiotic diffusion discs (Gentamicin from Alkom Laboratories LTD) were used as positive reference standards (10 μg/disc) for all bacterial strains.

#### Disc-diffusion assay

Petri plates (9 cm) were prepared with 20 ml of a base layer of molten Mueller Hinton agar (DIFCO, Becton Dickinson, USA). Each Petri plate was inoculated with 15 μl of each bacterial suspension (10^6^ CFU/ml). After drying in a sterile hood, 6 mm diameter discs soaked with 10 μl of the different dilutions of polyphenol-rich fractions were placed on the agar. Discs containing Gentamicin were used as positive controls and 10% DMSO was used as a negative control. The plates were incubated for 24 h at 37°C. The diameters of the inhibition zones were evaluated in millimeters. The fraction inducing inhibition zone ≥ 3 mm around disc were considered as antibacterial. All tests were performed in triplicate and the bacterial activity was expressed as the mean of inhibition diameters (mm) produced [13].

#### Minimum inhibitory concentration (MIC)

Minimum inhibitory concentration (MIC) was determined by the microdilution method in culture broth as recommended by [14]. Eight serial two-fold dilutions of polyphenol-rich fractions or conventional antibiotic (Gentamicin) were prepared as described before, to obtain final concentration range of 4 mg/ml to 62.5 μg/ml. The last wells (n°8) served as sterility controls (contained broth only) or negative control (broth + inoculums). The 96-well micro-plates (NUNC, Danemark) containing 100 μL of Mueller Hinton (MH) broth were used. For each bacteria strain, three columns of eight wells to the micro-plate were used. Each well has getting: the culture medium + polyphenol-rich fractions or Gentamicin + inoculum (10 μl of inocula) and INT (50 μl; 0.2 mg/ml for 30 min). The plates were covered and incubated at 37°C for 24 h. All tests were performed in triplicate and the bacterial activity was expressed as the mean of inhibitions produced. Viable microorganisms reduced the yellow dye to a pink colour. The MIC was defined as the lowest concentration of substance of polyphenol-rich fractions at which no colony was observed after incubation. So, the MIC was defined as the lowest concentration where no change was observed, indicating no growth of microorganism.

#### Minimal bactericidal concentration (MBC)

Minimum bactericidal concentration (MBC) was recorded as a lowest fraction concentration killing 99.9% of the bacterial inocula after 24 h incubation at 37°C. Each experiment was repeated at least three times. MBC values were determined by removing 100 μl of bacterial suspension from subculture demonstrating no visible growth and inoculating nutrient agar plates. Plates were incubated at 37°C for a total period of 24 h. The MBC is determined with the wells whose the concentrations are ≥ MIC [13,15]. The MBC were determined in Mueller Hinton (MH) agar (DIFCO, Becton Dickinson, USA) medium.

#### Evaluation of bactericidal and bacteriostatic capacity

The action of an antibacterial on the bacterial strains can be characterized with two parameters such as Minimum inhibitory concentration (MIC) and Minimum bactericidal concentration (MBC). According to the ratio MBC/MIC, we appreciated antibacterial activity. If the ratio MBC/MIC = 1 or 2, the effect was considered as bactericidal but if the ratio MBC/MIC = 4 or 16, the effect was defined as bacteriostatic [16].

#### Acute toxicity study in mice

Healthy male and female Swiss mice (20-30 g) were randomly divided into 6 groups (1 control group and 5 treated assay groups) of 6 animals (3 male and 3 female). They deprived of food (but with access to drinking water) for 15 h prior to the administration of the test suspension. The control group received water containing 10% dimethylsulfoxide (DMSO) administered by intraperitoneally. The aqueous extract acetone of *Sida urens* suspended in 15% DMSO was administered intraperitoneally at doses of 1800, 2000, 2500, 3000 and 6000 mg/kg. The general behaviour of the mice was observed at 120 min after the treatment. The animals were observed for morbidity and mortality once a day for up 14 days, with food and water *ad libitum*. The number of survivors after the period of 14 days was noted. The toxicological effect was assessed on the basis of mortality for 14 days, which was expressed by the median lethal dose value (Lethal Dose 50 or LD50) estimated from the regression of log-probit mortality rate [17].

#### Analgesic capacity

**Acetic acid-induced writhing test.** The analgesic activity of the samples was studied using acetic acid-induced writhing model in mice model [18]. Nociception was induced by an intraperitoneal injection of 0.6% acetic acid solution in a value of 10 ml/kg body weight. The animals were divided into five groups with six mice in each group. Group I, animals received vehicle (10% DMSO in water, 10 ml/kg body weight), animals of group II received paracetamol 100 mg/kg body weight while animals of group III; group IV and group V were treated with 100; 200 and 400 mg/kg body weight of polyphenol-rich fractions dissolved in 10% DMSO 1 h orally before acetic acid injection. The number of writhes occurring between 5 and 20 min after acetic acid injection was recorded. The analgesic effect was expressed as the percentage reduction of writhes in treated mice compared to those in the control group. The percentage inhibition was calculated using the following equation 1:

$$\left(\%\right)\;\mathrm{inhibition}\;=\left(A-B/A\right)\times 100$$

Where A is mean for the control group and B is mean for the treated group.

#### Statistical analysis

The data were expressed as Mean ± Standard deviation (SD) of six determinations (n = 6). Results were analyzed by one-way ANOVA followed by Dunnett’s *t*-test using Prism 4 software. The level of significance was accepted at p ≤ 0.05.

## Results

### Antibacterial capacity

In this present study, six bacteria strains were used. The antibacterial assays were performed by the agar-well diffusion and the broth micro dilution methods; so that they could be qualified and quantified by inhibition zone diameters, MIC and MBC. One noticed that the susceptibility of the bacteria to the polyphenol-rich fractions on the basis of inhibition zone diameters varied according to the microorganism. There is a significant variation in the diameters of inhibition zone values (DIZ) of polyphenol-rich fractions (Table 1).

**Table 1:** **The diameters of inhibition zone of polyphenol-rich fractions from *Sida urens *L. and Gentamicin**

**Microorganisms**	**Polyphenols (mm)**	**Gentamicin (mm)**
***Pseudomonas aeruginosa***	-	23.67 ± 1.00
***Streptococcus salivarius***	20.33 ± 0.58	19.00 ± 0.58
***Streptococcus viridans***	19.00 ± 1.00	-
***Streptococcus mutans***	18.67 ± 2.08	-
***Bacillus megaterium***	20.33 ± 1.53	23.00 ± 1.00
***Neisseria catarrhalis***	16.66 ± 1.15	23.66 ± 1.53

The results are the means of number of the colonies ± standard deviations.

(−) = Resistant.

As for the micro-well dilution assay (MIC) and Minimum bactericidal concentration (MBC) of polyphenol-rich fractions, result varied according to the microorganism (Table 2 and Table 3). The MIC values were ranged from 62.5 to 250 μg/ml and for the MBC values were ranged from 125 to 1000 μg/ml. The bactericidal and bacteriostatic effect of polyphenol-rich fractions was determined using the ratio MBC/MIC (Table 3).

**Table 2:** **Minimum Inhibitory Concentration (MIC) of polyphenol-rich fractions from *Sida urens *L. and Gentamicin**

**Microorganisms**	**Polyphenols (μg/ml)**	**Gentamicin (μg/ml)**
*Pseudomonas aeruginosa*	nd	31.25
*Streptococcus salivarius*	62.5	125
*Streptococcus viridans*	125	nd
*Streptococcus mutans*	125	nd
*Bacillus megaterium*	62.5	31.25
*Neisseria catarrhalis*	250	62.5

The results are the means of number of the colonies ± standard deviations.

nd: No detected activity.

**Table 3:** **Minimal bactericidal concentration (MBC) of polyphenol-rich fractions from *Sida urens *L., and their bactericidal and bacteriostatic effect (MBC/MIC)**

**Microorganisms**	**MBC (μg/ml)**	**MMBC/MIC**	**Effect**
*Pseudomonas aeruginosa*	nd	nd	nd
*Streptococcus salivarius*	125	2	+
*Streptococcus viridans*	250	2	+
*Streptococcus mutans*	500	4	-
*Bacillus megaterium*	125	2	+
*Neisseria catarrhalis*	1000	4	-

The results are the means of number of the colonies ± standard deviations.

+: bactericidal effect, - bacteriostatic effect, nd: no detected activity.

### Acute toxicity study

The *Sida urens* aqueous acetone extract employed for acute toxicity studies were found to be non-toxic. The value of LD_50_ is greater than 5000 mg/kg. No significant difference in body weight gain of the treated assay groups over the period of observation.

### Analgesic capacity

#### Acetic acid-induce writhing test

As for acetic acid-induced writhing test, the fraction effectively reduced the number of abdominal muscle contractions induced by 0.6% acetic acid solution. The fraction has a dose- dependent protection. Results are presented in the Table 4. The polyphenol-rich fractions at different doses produced significantly effects compared to the control group (p < 0.001) and a dose-dependent anti-nociceptive activity.

**Table 4:** **Effect of polyphenol-rich fractions from *Sida urens *L. on writhing-induced by acetic acid**

**Compounds**	**Doses (mg/kg b.w.)**	**Number of writhing**	**Inhibitions (%)**
Control	- - - - - - - - - - - - - - - - - - -	58.10 ± 1.10	- - - - - - - - - - - - - - - - - - - - -
Paracetamol	100	26.00 ± 1.10^***^	55.24
AAE _*Sida urens*_	100	22.18 ± 0.41***	61.82
AAE _*Sida urens*_	200	17.24 ± 0.10***	70.32
AAE _*Sida urens*_	400	10.02 ± 0.68***	82.75

Values are Mean ± SEM (n = 6) one-way ANOVA Followed by Dunnett’s t-test: Compare all vs. Control group (reference drug): **P < 0.01; ***P < 0.001 compared with control.

## Discussion

Nowadays the degree of dental caries and related problems are increasing with severe effects. As part of search for effective phytoderivatives against dental pathogens, *Sida urens* L., was collected and its fraction was applied against bacterial isolate. The result evidenced that *Sida urens* is with acceptable levels of bactericidal activity and showed good antibacterial activity as compared with that of standard drug Gentamicin.

The bacteriostatic and bactericidal activity could be ascribed to the presence of flavonoids and coumarin-related compounds. A probable degree of lipophilicity might be responsible for the polyphenol-rich fractions being higher in activity than Gentamicin. Lipophilicity toxicity is due to the interactions with the membrane constituents and their arrangement [19]. The reason for the different activity between Gram-positive and Gram-negative bacteria could be accounted for by the morphological differences between these micro-organisms. Gram-negative bacteria have an outer phospholipidic membrane carrying the structural lipopolysacharide components. This makes the cell wall impermeable to lipophilic solutes while porins constitute a selective barrier to the hydrophilic. The Gram-positive bacteria become therefore more susceptible having only an outer peptidoglycan layer which is not an effective permeability barrier [20]. Generally therefore, Gram-negative bacteria are more resistant than Gram-positive bacteria because of the complexity of the cell wall of Gram-negative bacteria [21]. Results of antibacterial activity confirm again that the plant extracts inhibited the Gram-positive bacteria better than the Gram-negative ones. Moreover, the reason could be attributed to the presence of extra outer membrane in their cell wall acting as barrier for the compound(s) to diffuse into the bacterial cells [22]. The findings demonstrated promising antibacterial activity of polyphenol-rich fractions from *Sida urens*. The zones of inhibition and bactericidal and bacteriostatic effect values suggest that this Malvaceae can significantly inhibit bacterial growth in a laver dose dictates its potential as a source of active chemicals that might be used for the discovery of new antibacterial agent. We presume that the presence of the natural antimicrobial compounds such as phenolic compounds from the plant. In effect, phenolic compounds as simple phenols and phenolic acids whose mode of action is via enzyme inhibition by the oxidized compounds, flavones, flavonoids and flavonols have the ability to complex with proteins and bacterial cell walls and tannins inactivate microbial adhesions, enzymes and cell envelope transport proteins [23]. Therefore, the presence of flavonoids, phenolic in plants has been shown to be responsible for antimicrobial activity in plants [20]. Their role is to protect plants against microbial or insect damage [23]. Since medicinal plants contain pharmacologically active substances with antimycobacterial, antibacterial and antifungal properties [24], the antibacterial activity of the plants tested could therefore be attributed to the presence of these compounds.

The results of the present study indicated that the extract of *Sida urens* is not poisonous. During the 14 day period of acute toxicity evaluation, some signs of toxicity were observed, but they were all quickly reversible. Pharmacological substances whole LD_**50**_ is less than 5 mg/kg body weight are classified in the range of highly toxic substances, those with a LD_**50**_ between 5 mg/kg body weight and 5000 mg/kg body weight are classified in the range of moderately toxic substances and those with the lethal dose is more than 5000 mg/kg body weight not toxic. In this fact, if we refer to this classification we could say that the extract of *Sida urens* is not toxic and would be regarded as being safe [25].

The analgesic activity study revealed that polyphenol-rich fractions showed good activity compared with that of standard drug paracetamol. Acetic acid induced writhing in mice attributed visceral pain finds much attention of screening analgesic drugs [26]. The polyphenol-rich fractions showed significant analgesic action compared to the reference drug. Pain sensation in acetic acid induced writhing method is elicited by triggering localized inflammatory response resulting release of free arachidonic acid from tissue phospholipid [27] via cyclooxygenase (COX) and prostaglandin biosynthesis [28]. In order words, the acetic acid induced writhing has been associated with increased level of PGE2 and PGF2α in peritoneal fluids as well as lipoxygenase products [29]. The increase in prostaglandin levels within the peritoneal cavity then enhances inflammatory pain by increasing capillary permeability [30]. The acetic acid induced writhing method was found effective to evaluate peripherally active analgesics. The agent reducing the number of writhing will render analgesic effect preferably by inhibition of prostaglandin synthesis, a peripheral mechanism of pain reduction [28]. The significant pain reduction of polyphenol-rich fractions might be due to the presence of analgesic principles acting with the prostaglandin pathways. The abdominal writhing induced by acetic acid was also reported to be less selective [31] and proposed to act indirectly by releasing endogenous mediators stimulating neurons that are sensitive to other drugs such as narcotics and centrally acting agents [32].

Therefore, it is assumed that these compounds may be responsible for the observed analgesic activity. Flavonoids were reported to have a role in analgesic activity primarily by targeting prostaglandins [33]. There are also reports on the role of tannins in anti-nociceptive activity [34].

## Conclusion

The results of present study supports the traditional usage of the *Sida urens* and suggests that this plant possesses compounds with high antibacterial and analgesic properties that can be used as antibacterial and analgesic agents in developing new drugs for the therapy of dental caries bacteria. Further purification and characterization of the active principles from the effective extracts will provide a better understanding of the antimicrobial and analgesic mechanisms. This objective will be achieved in parallel with the investigation on more pathogenic agents currently going on.

## Competing interests

The authors declare that they have no competing interests.

## Authors’ contributions

KK, MO and CID carried out the study and wrote the manuscript, PZ, AH, OS, MB and NB supervised the work, the manuscript and contributed to the manuscript corrections. All authors read and approved the final manuscript.
